# “Dysrationalia” Among University Students: The Role of Cognitive Abilities, Different Aspects of Rational Thought and Self-Control in Explaining Epistemically Suspect Beliefs

**DOI:** 10.5964/ejop.v15i1.1696

**Published:** 2019-02-28

**Authors:** Nikola Erceg, Zvonimir Galić, Andreja Bubić

**Affiliations:** a Department of Psychology, Faculty of Humanities and Social Sciences, University of Zagreb, Zagreb, Croatia; b Department of Psychology, Faculty of Humanities and Social Sciences, University of Split, Split, Croatia; University of Belgrade, Belgrade, Serbia

**Keywords:** dysrationalia, epistemically suspect beliefs, cognitive abilities, rational thinking, self-control

## Abstract

The aim of the study was to investigate the role that cognitive abilities, rational thinking abilities, cognitive styles and self-control play in explaining the endorsement of epistemically suspect beliefs among university students. A total of 159 students participated in the study. We found that different aspects of rational thought (i.e. rational thinking abilities and cognitive styles) and self-control, but not intelligence, significantly predicted the endorsement of epistemically suspect beliefs. Based on these findings, it may be suggested that intelligence and rational thinking, although related, represent two fundamentally different constructs. Thus, deviations from rational thinking could be well described by the term “dysrationalia”, meaning the inability to think rationally despite having adequate intelligence. We discuss the implications of the results, as well as some drawbacks of the study.

Recently, there was an interesting story trending in Croatian media. It was about a well-known local movie director that sued a clairvoyant and tarot master. Despite spending large amount of money on consultations, neither telephone nor tarot sessions helped the director. Therefore, she decided to sue the psychic because the psychic was obviously a fraud. Approximately at the same time, a relatively famous Serbian football player came out with the devastating story about black magic spells being cast on his family. However, this one had a happy ending: after they found a striped shirt and burned what was on the inside, exactly what a psychic told him and his family to do, the things luckily went back to normal.

Although these stories are extreme examples of irrational behavior, most of us probably know some people that, despite being sufficiently intelligent, nevertheless hold weird or irrational beliefs. Stanovich (2009) coined the term „dysrationalia” to describe the inability to think and behave rationally despite having adequate intelligence. Similar to the examples in the introduction, the irrational beliefs often take form of the epistemically suspect beliefs (ESB). ESB can be defined as beliefs that are in conflict with common naturalistic conceptions of the world (Pennycook, Cheyne, Barr, Koehler, & Fugelsang, 2015a). The main goal of this study was to investigate the relationship between this kind of beliefs and rational thinking.

## Rational Thinking and Cognitive Abilities: Definition and Measurement

By looking at the above definition of the term „dysrationalia”, it is clear that some authors hold the view that cognitive abilities, i.e. intelligence, and the ability to think rationally are not the same thing. Moreover, they argue that rational thinking abilities are a broader concept than the cognitive abilities, a construct that encompasses intelligence, but also some other characteristics such as reflection, open-minded thinking etc. (Stanovich 2009, 2014; Stanovich & West, 2008; Stanovich, West, & Toplak, 2016).

There are at least two positions from which it can be argued for a separation of rational thinking and cognitive abilities. The first set of arguments comes from the tripartite theory of mind that differentiates between autonomous, algorithmic and reflective parts of the mind (Pennycook, Fugelsang, & Koehler, 2015b; Stanovich, 2009; Stanovich et al., 2016). This theory is a direct expansion of the dual-process theory that differentiates between the System 1 and System 2 (Evans & Stanovich, 2013). Whereas autonomous, fast, parallel, non-conscious, and automatic processing of information characterize System 1, System 2 is more serial, conscious, rule-based, abstract and working memory dependent. However, it seems that it is possible to differentiate between two different aspects of System 2 processing, the algorithmic and the reflective mind. Now relatively famous, the bat-and-ball problem from the Cognitive Reflection Test (CRT; Frederick, 2005) elegantly illustrates this. Consider the following problem: „A bat and a ball cost $1.10 in total. The bat costs $1 more than the ball. How much does the ball cost?“ The peculiarity of this, as well as other problems from the test, is that they automatically trigger relatively strong initial response (i.e., 10 cents). However, after a more careful reflection, it is clear that this is an incorrect answer, and that the right response is in fact 5 cents. Thus, in order to overcome the initial wrong response (generated by the autonomous mind), and arrive to the correct one, one has to first reflect on the answer and recognize the need to engage in a more deliberate processing (the reflective mind), but also to possess adequate computational power, knowledge and abilities to calculate the right answer (algorithmic mind). By looking at this example, it becomes clear that what is generally assessed by IQ tests mostly taps into the operations of the algorithmic mind, but not the reflective mind. Therefore, in order to think rationally, the adequate computational power (e.g. intelligence) is necessary, but not sufficient: the willingness to reflect on one's initial responses and engage in more deliberate thinking seems to be the key factor here (Pennycook et al., 2015b).

The second position from which it is possible to argue for a separation of rational thinking and cognitive abilities relies on the definition of rationality. Namely, there appears to be two kinds of rationality: epistemic and instrumental rationality (Stanovich et al., 2016). The first one, epistemic rationality, refers to the correctness of the beliefs, i.e. “how well beliefs map onto the actual structure of the world” (Stanovich, West, & Toplak, 2017, p. 205). For our beliefs to be rational, they must be true, or correspond to the objective state of the world. The second type of rationality, instrumental rationality, refers to „behaving in the world so that you get exactly what you most want, given the resources (physical and mental) available to you“ (Stanovich et al., 2017, p. 205). Thus, it is said that epistemic rationality is about what is true and instrumental rationality is about what to do. However, these two types of rationality are interrelated. Namely, to be able to take actions that fulfill our goals (instrumental rationality), we need to base those actions on beliefs that are properly matched to the world (epistemic rationality; Stanovich, 2014). The key argument here is that conventional IQ tests, while measuring the computational power of the algorithmic mind (Stanovich, 2012), do not capture the two types of rational thinking, and that different tasks should be developed in order to measure one’s rational thinking abilities.

Given that the conventional IQ tests do not properly assess the rational thinking abilities, what kind of tasks would be appropriate for measuring these kinds of abilities? As it follows for the previous argumentation, one of the frameworks for the classifications of these tasks could be along the two dimensions: a) which of the two types of rationality the task is supposed to assess; b) in what degree does the performance on the task depend on the reflective versus algorithmic mind. For example, previously discussed CRT (Frederick, 2005) is widely used in judgment and decision literature as it clearly taps into the functioning of the reflective mind, while being somewhat less demanding for the algorithmic mind (requiring certain amount of computational power and only the knowledge of basic algebra that most of the people possess).

Furthermore, other tasks have been used in judgment and decision making literature that tap into epistemic and instrumental rationality. On the one hand, some aspects of epistemic rationality include the ability to make coherent probability judgments, resistance to overconfidence in judgments, understanding of chance events, and consideration of the alternative hypothesis when evaluating evidence (Stanovich, 2014). Thus, these kinds of tasks are used to assess the epistemic rationality. On the other hand, among key aspects of instrumental rationality is the ability to make judgments and decisions without the influence of irrelevant context. For example, one cannot make good decisions in their own interest if those decisions are influenced by irrelevant inputs such as frames, defaults, vividness of stimuli or irrelevant affect (Stanovich, 2014). Thus, the tasks that measure judgments free from these irrelevant facts are often used as measures of instrumental rationality.

There are currently no developed and widely used measures of rational thinking that are based on the described theoretical consideration. Perhaps the most ambitious one is the Comprehensive Assessment of Rational Thinking battery (CART; Stanovich et al., 2016). However, it still represents work-in-progress, and in its current form, with over 100 items, seems cumbersome for both researchers and participants. Therefore, the rational thinking measures in most of studies are often developed ad-hoc, by lumping together several categories of the previously described items (e.g. Pennycook, Ross, Koehler, & Fugelsang, 2016; Toplak, West, & Stanovich, 2011, 2017).

These rational thinking measures generally show two important characteristics. First, their correlation with intelligence is generally either low or non-existent (Stanovich, 2009; Stanovich & West, 2008, 2014; Stanovich, West, & Toplak, 2013, 2016). Second, they are able to predict a wide range of real-life outcomes. For example, low scores on these tasks have been shown to predict physicians choosing more often suboptimal medical treatments, people failing to accurately assess risks in their environment, parents failing to vaccinate their children, the amount of money wasted on questionable medical remedies, as well as holding more ESB (Pennycook et al., 2015a; Pennycook, Cheyne, Seli, Koehler, & Fugelsang, 2012; Pennycook et al., 2015b; Pennycook et al., 2016; Stanovich & West, 2014).

## Epistemically Suspect Beliefs, Cognitive Abilities and Rational Thinking

As noted before, the ESB are the main topic of interest in this paper. Although there are different types of those kind of beliefs (e.g. paranormal, supernatural, magical, superstitious, and even religious beliefs), it seems that they all share one common characteristic - ontological confusion. More precisely, these beliefs involve category mistakes, such that the core ontological distinction between the categories of physical, biological and psychological phenomena are blurred (Lindeman & Svedholm, 2012). Some of the examples would be thoughts that can move objects, existence of mind independently of bodies or sense of purpose in chance events. Lindeman and Svedholm (2012) conclude that all these different beliefs are not fundamentally different, which is corroborated by Lindeman and Aarnio's (2006) findings demonstrating that all these beliefs can be explained by one higher-order factor.

Recently, Risen (2016) discussed superstitious thinking within the dual-process framework. However, given the above discussion, different types of ESB can probably be accommodated within that same framework. Specifically, in line with the default-interventionist model (Kahneman & Frederick, 2005), she proposed that System 1, due to its features such as reliance on heuristics and attribute substitution, causal intuitions and confirmation bias, generates superstitious intuitions quickly and easily and that these intuitions then serve as a default for judgment and behavior. System 2 may or may not correct the initial intuition. Features of System 1 such as representativeness and availability heuristics, causal intuitions or conformation bias work together in supporting the superstitious intuitions. Certain characteristics of System 2 such as the motivation and the ability to be rational increase the probability that the System 2 will engage and override the initial intuition.

When represented in the tripartite theory of mind framework, System 2 refers to the rational mind and consists of both the algorithmic and reflective mind. Therefore, based on the Risen’s theory, it can be hypothesized that ESB will especially be prominent in people with lower abilities of algorithmic and reflective mind. As postulated previously, algorithmic mind can be assessed with a cognitive ability test, while the reflective mind should be assessed with a battery of different tasks that capture instrumental and epistemic rationality (i.e. rational thinking tasks). However, besides these types of tasks, reflective mind is often assessed by so called thinking dispositions or cognitive styles (e.g. Aarnio & Lindeman, 2005; Lindeman & Svedholm-Häkkinen, 2016; Pennycook et al., 2015a; Stanovich et al., 2016).

Although several models and theories of cognitive styles were proposed (e.g. Allinson & Hayes, 1996; Dewberry, Juanchich, & Narendran, 2013; Scott & Bruce, 1995), one of the best known and widely used theory is Epstein’s (1991) Cognitive-experiental self-theory. This theory draws directly from dual-process theory and posits two independent styles, analytic and intuitive one. Analytical style, emphasizing a conscious, analytical thinking approach, is often measured by the Need for Cognition Scale (NFC; Cacioppo & Petty, 1982), while the intuitive style is assessed with the Faith in Intuition Scale (FI). These two scales represent subscales of the Rational – Experiental Inventory (Epstein, Pacini, Denes-Raj, & Heier, 1996; Norris, Pacini, & Epstein, 1998). Previous research has shown that analytical cognitive style was negatively related to ESB (Lindeman & Aarnio, 2006; Lindeman & Svedholm Häkkinen, 2016; Pennycook et al., 2012), while the intuitive style was positively related with the ESB (Aarnio & Lindeman, 2005; Sadler-Smith, 2011).

In addition to that, we hypothesized that the rational thought could also be aided by self-control. Self-control is widely regarded as a capacity to change and adapt the self to produce a better, more optimal fit between self and world (Tangney, Baumeister, & Boone, 2004), which resembles Stanovich et al. (2016) notion of epistemic rationality. In a similar fashion as the System 2 which overrides initial intuitions, self-control is defined as the capacity to alter or override dominant response tendencies and to regulate behavior, thoughts and emotions (de Ridder, Lensvelt-Mulders, Finkenauer, Stok, & Baumeister, 2012). One of the ways in which self-control could operate is through the education, where those with high self-control should be more successful as they are probably better at getting tasks done on time, using study time effectively, choosing appropriate courses, etc. (Tangney et al., 2004). Regardless of the mechanisms of the self-control operation, if self-control could enhance one’s rationality of thinking and behaving, it would be possible for it to have the effect of suppressing the ESB.

## Our Study

The main goal of this study is to explore the role that different aspects of rational thought play in ESB. More precisely, we aimed to compare the validity of different measures of rational thought encompassing measures of cognitive abilities, reflective thinking abilities and cognitive styles in their capacity to predict variance of ESB measures. We therefore postulated five hypotheses. First, we expected that both cognitive abilities and rational thinking abilities, as components of rational thought (i.e. algorithmic and reflective mind), will be negatively related to ESB. Furthermore, based on the previous findings, we expected a negative correlation between the analytical cognitive style and the ESB, and a positive correlation between the intuitive cognitive style and the ESB. Finally, based on the previous description of self-control, we expected it to be negatively correlated with the ESB.

## Methodology

### Participants and Procedure

A total of 159 students of Faculties of humanities and social sciences from Zagreb and Split (129 females and 29 males), aged 18 – 26 (*M* = 21.30; *SD* = 1.50) participated in this study. They were given a battery of tasks and scales in a classroom setting, and they all received course credits for their participation, regardless of their performance on the tests. Due to the questionnaires being in a paper and pencil format, order of the questionnaires and tests was fixed and same for all the participants. Cognitively more demanding tasks were presented first (cognitive ability test was always the first one, followed by the cognitive reflection test, rationality tasks and syllogistic reasoning tasks), followed by the self-report measures (i.e. cognitive styles and self-control).

### Instruments

*ESB* were assessed using items from Toplak et al. (2011). The scale consisted of 13 items in total, assessed on the five-point scale (Cronbach α = .75). The total score was calculated as a mean score of all the items, meaning that it could take any value between one and five. The items tapped into four different concepts: superstitious beliefs (e.g. “*When something good happens to me, I believe it is likely to be balanced by something bad*.”), luck (e.g. “*I have personal possessions that bring me luck at times.*”), paranormal beliefs (“*Astrology can be useful in making personality judgments.*”), and extrasensory perception (“*Dreams can provide information about the future.*”).

*Cognitive ability* was assessed using Advanced Progressive Matrices (APM; Raven, Court, & Raven, 1988). The instrument consists of 36 items overall, so a total individual score can be any number between 0 and 36. Participants had 30 minutes to solve the test, and it was always the first test in the battery.

*Rational thinking abilities* were assessed with three different types of tasks:

a) *Cognitive Reflection Test* (CRT; Frederick, 2005) is an instrument used for measuring individuals’ ability to resist reporting an intuitive incorrect answer and to calculate and report the correct one. It consists of three items (Cronbach α = .70), with the total score representing the proportion of correctly answered items.

b) *Rationality tasks* (Toplak et al., 2011) were designed to mostly tap into either instrumental or epistemic rationality. For example, instrumental rationality was assessed mainly with different tasks emphasizing the ability to disregard irrelevant contextual factors when making judgments and decisions (i.e., tasks that measure the ability to resist framing effects, sunk costs, outcome bias or being affected by vivid stimuli). For example, in the framing task, we captured whether an individual’s choice was affected by the problem being framed in terms of gains or losses. If one’s choices were consistent irrespectively of the frame, it would mean that one is generally more able to resist the irrelevant context when making judgments and, therefore, that one is more rational. On the other hand, epistemic rationality was assessed with tasks such as those related to gambler’s fallacy, covariation detection, base rate neglect, denominator neglect etc. For example, in the context of the gambler’s fallacy task, if a person would realize that the outcomes of chance events (such as rolling a dice) cannot be influenced by previous outcomes, it would suggest that that person in more rational. There were 11 tasks in total (Cronbach α = .44). The total score on these 11 tasks was calculated as a proportion of correct answers, and similarly as with the CRT, it was in range between 0 and 1.

c) *Syllogistic reasoning tasks* (Toplak et al., 2011) measure susceptibility to belief bias. They pit the believability of a conclusion against its logical validity. In that regard they are somewhat similar to the CRT tasks in that, in order to arrive at the correct conclusion, a person must first notice that the believable conclusion, although intuitively receptive, is false. The total score, similarly as with the CRT and rationality tasks was a proportion of correct answers given on these five tasks (Cronbach α = .62).

Finally, a Rational Thinking Ability (RTA) score was calculated by combining the score of these three types of tasks into one composite score. This approach was previously used by a number of researchers in the field of individual differences in decision making (e.g. Barr, Pennycook, Stolz, & Fugelsang, 2015; Pennycook et al., 2016; Toplak et al., 2011; West, Toplak, & Stanovich, 2008). The main rationale behind combining these three constructs into a composite score is that all of the items/constructs represent some component of rational thinking (Stanovich et al., 2016). Specifically, the rational thinking ability score was calculated by averaging the scores on the three different types of tasks. Therefore, a person could score anywhere between a 0 and 1 on the rational thinking ability. When calculating the total score in this way, the internal consistency coefficient for this measure containing three items was Cronbach α = .59^i^. Relatively low reliability coefficients in this field are not uncommon. As Stanovich et al. (2017) put it, “rationality is multifarious concept, it is unlikely to yield as substantial a g-factor as is the case with intelligence” (p. 216).

However, it is important to note that Cronbach’s alpha can underestimate reliability, especially when applied to very short measures of broad constructs (Sandy, Gosling, Schwartz, & Koelkebeck, 2017), since the alpha is a function of a number of items in a scale and average correlations among them. Since there are only three items combined in this composite score, the low alpha coefficient is not surprising. On the other hand, another index of internal consistency, *omega*, which is deemed to be a more sensible index of internal consistency (Dunn, Baguley, & Brunsden, 2014), yields higher estimate of reliability of ω = .67. Furthermore, inter-correlations among the three measures are all medium to high (lowest correlation is *r* = .32 between syllogistic reasoning and CRT), which also suggests that Cronbach’s alpha probably underestimates the real reliability in this case.

*Analytical cognitive style* was measured by the adapted Need for Cognition Scale (NFC; Cacioppo & Petty, 1982) and consists of five items (e.g., *“I would prefer complex to simple problems”*; Cronbach α = .84). On the other hand, Faith in Intuition Scale (FI) is used for measuring the *intuitive cognitive style*, and also consists of five items overall (e.g., *“My initial impressions of people are almost always right”*; Cronbach α = .83). The participants were instructed to assess how well each of the items describes themselves using five point ratings (*1 = not at all* to *5 = very much*). The total score on both scales was calculated as average assessment over five items. Thus, on both scales a person could score anywhere between 1 and 5.

*Self-control* was measured with the Brief Self-Control Scale (BSC; Tangney et al., 2004). The BSC is designed for measuring self-control, or the ability to control one's instincts, emotions, thoughts, habits and behavior. It consists of 13 items (e.g., *“I wish I had more self-discipline”*; Cronbach α = .83) and the participants were instructed to indicate, using five point ratings (*1 = not at all* to *5 = very much*), how well each of the items describes how they typically behave and feel. Similarly as with NFC and FI, the total score was calculated as average assessment over thirteen items. Thus, an individual score could be anywhere between 1 and 5.

## Results

As seen from the relatively low mean for the ESB score in the Table 1, college students are a relatively non-superstitious group. On the other hand, they seem to be highly intelligent as a group, with most individuals having above average intelligence score. To examine the relationship between the ESB on the one hand, and rational thinking ability, analytical and intuitive cognitive styles and self-control on the other hand, we calculated the correlation coefficients.

**Table 1:** Descriptive Statistics and Reliability Coefficients of the Measures Used in the Study

Measure	*M*	*Mdn*	*SD*	Min	Max
ESB					
ESB scale	1.92	1.77	0.59	1	4.15
Cognitive abilities					
APM	27.24	28	4.50	14	36
Rational thinking abilities					
CRT	0.58	0.67	0.39	0	1
Rationality tasks	0.67	0.64	0.18	0.09	1
Syllogisms	0.69	0.60	0.28	0	1
RTA	0.66	0.67	0.22	0.10	1
Cognitive styles					
NFC	3.79	3.80	0.76	1.40	5
FI	3.33	3.40	0.73	1	5
Self-control					
BSC	3.05	3.00	0.61	1.46	5

*Note.* ESB = Epistemically Suspect Beliefs; APM = Advanced Progressive Matrices; CRT = Cognitive Reflection Test; RTA = Rational Thinking Abilities; NFC = Need for Cognition; FI = Faith in Intuition; BSC = Brief Self-control Scale.

The correlational analysis indicated that the ESB were significantly correlated with all the variables except for the cognitive abilities (*r* = -.04). Specifically, ESB were negatively correlated with the Rational Thinking Ability (RTA) score (*r* = -.18), the Need for Cognition (NFC) score (*r* = -.23) and the self-control score (*r* = -.16), while they were positively correlated with the Faith in Intuition (FI) score (*r* = .35). The correlations between all the variables are presented in the Table 2, while scatter plots of their relationships and their distributions are seen in Figure A in the Appendix.

**Table 2:** Correlation Coefficients Between the Assessed Variables

Variable	1	2	3	4	5	6
1. ESB	–					
2. APM	-.04	–				
3. RTA	-.18*	.43**	–			
4. NFC	-.23**	.14	.24**	–		
5. FI	.35**	.00	-.08	-.02	–	
6. Self-control	-.16*	-.14	-.19*	.12	.01	–

*Note.* ESB = Epistemically Suspect Beliefs; APM = Advanced Progressive Matrices; RTA = Rational Thinking Ability; NFC = Need for Cognition; FI = Faith in Intuition.

*p < .05. **p < .01.

As seen in the Table 2, the correlation between the APM score and rational thinking abilities score was *r* = .43, which could be classified as a medium sized correlation. Contrary to that, the correlation between APM and cognitive styles was much lower and non-significant. These effect sizes are pretty similar to those generally observed in the literature (e.g. Stanovich et al., 2016).

Next, to identify variables that serve as unique predictors of ESB, as well as to assess how much of the variance in those beliefs can be explained by the variables included in the model, we conducted a regression analyses. The results of this analysis are presented in the Table 3.

**Table 3:** Regression Analysis With Cognitive Abilities, Rational Thinking Abilities, Analytic Cognitive Style, Intuitive Cognitive Style and Self-Control as Predictors, and Epistemically Suspect Beliefs Score as an Outcome

Predictors	β	Dominance coefficients
Cognitive abilities	.04	0.00
Rational thinking abilities	-.17*	0.03
Analytic cognitive style	-.18*	0.04
Intuitive cognitive style	.33**	0.11
Self-control	-.16*	0.02

*Note.* *R* = .45; *R*^2^ = .21**; Adj. *R*^2^ = .18**; *F*(5, 147) = 7.62**.

*p < .05. **p < .01.

As can be seen from the Table 3, the model explained overall 21% (corrected 18%) of the variance in the ESB. As expected from looking at the correlation coefficients, cognitive abilities did not predict ESB, being the only non-significant predictor in the model. On the other hand, higher scores on the rational thinking abilities, analytical cognitive style and self-control predicted lower susceptibility to ESB. Contrary to that, higher score on the intuitive cognitive style predicted higher susceptibility to ESB.

In addition to regression analysis, we also conducted the dominance analysis (Azen & Budescu, 2003; Budescu, 1993; LeBreton & Tonidandel, 2008) that can quantify the relative importance of each of the predictors for the outcome. Specifically, we calculated the general dominance coefficient for each of the predictors. This is done by averaging the added variance explained by the predictor in all possible subsets of regression models. For example, if we wanted to calculate the general dominance coefficient for the predictor X1 in the model with three predictors, we would first calculate the proportion of variance explained by that predictor in all possible scenarios: when there are no additional predictors in the model besides X1, when there is additional predictor X2, when there is additional predictor X3 and when all three predictors (X1, X2 and X3) are in the model. Finally, we would average all calculated Δ*R^2^* and that number would represent the general dominance coefficient for that predictor. This coefficient has several important advantages over beta coefficients. For example, it is more intuitive since it represents the amount of variance explained in the outcome by each of the predictors in the model. Also, the sum of the general dominance coefficients of all the predictors in the model equals the total amount of variance in the outcome explained by the model. By inspecting the general dominance coefficients (Table 3), it is clear that cognitive abilities were irrelevant in explaining ESB. On the other hand, the intuitive cognitive style was by far the most important predictor of ESB, explaining over 50% of the total variance explained by the model.

Finally, as an additional, exploratory analysis, we tested for possible interactive effects of the individual differences in cognitive abilities, rational thinking and cognitive styles on ESB^ii^. For example, Čavojová (2016) showed that there was an interactive effect of cognitive abilities and thinking dispositions on ESB. To test for this possibility, we conducted three separate regression analyses. Cognitive ability (APM) was included as a predictor in every analysis, while RTA, NFC and FI were included in one of the analysis each. In the second step of every analysis, we included the interactive term of the predictors (APMxRTA, APMxNFC and APMxFI). The results showed that, contrary to Čavojová’s (2016) findings, neither of the interactions were significant and neither was able to explain additional portion of the variance above the effects of the individual predictors. Therefore, it seems that the effect of cognitive abilities on ESB does not depend on the level of the individual’s rational thinking ability or preferred cognitive style.

## Discussion

Probably the biggest contribution of our study follows from the use of a wide range of instruments for assessment of different aspects of rational thought. This aspect of study design allowed us to draw conclusions about relations among different aspects of the rational thought with ESB. What we found is that, somewhat surprisingly, intelligence could not predict extent to which individuals hold ESB. Contrary to that, other constructs that underpin rational thought, rational thinking ability, cognitive styles and self-control, were all independent predictors of ESB. On the one hand, the greater the rational thinking abilities, propensity for analytical thinking and self-control, the lesser the probability of a person endorsing ESB. On the other hand, greater propensity for intuitive thinking was related with holding more ESB. Both the regression analysis and supplementary dominance analysis indicated that inclination towards intuitive thinking is the most important predictor of all assessed within this study.

These results somewhat corroborate Risen’s (2016) model in which ESB arise from the heuristical and intuitive System 1 and thrive unless detected and overridden by the System 2. Assuming this model of ESB is correct, it is not surprising that the tendency for intuitive thinking was the most powerful predictor of endorsing these beliefs. Similarly, it is not surprising that various aspects of rational thought acted in another way, deterring those beliefs. Therefore, what can be concluded from this study is that the ESB are the result of some kind of interplay between the System 1 and System 2 processes. For example, in accordance with the parallel-competitive model, it could be that the two systems are activated at the same time and are constantly competing for an influence on the final response. Or it could be that, according to a default-interventionist model, System 1 generates the default intuitive response, but its expression depends on whether or not System 2 will intervene to correct it (see Evans, 2007, for a discussion about different models of a System 1/System 2 conflict resolution). However, it is important to note that, whatever the cognitive underpinnings of conflict resolution may be, greater disposition towards rational thinking would always be expected to result in lower ESB.

Although it was somewhat expected that the components of rational thought will be negatively related with the propensity to hold ESB, the puzzling finding of this research is the complete irrelevance of intelligence for these beliefs. Even from the laypeople point of view, one would expect that the smarter people would be less prone to foolish beliefs. Also, in the model proposed by Stanovich and associates (2016), intelligence is one of the essential parts of the rational thought and, in the cases when rational thought goes astray, it can be an indication of why that happened. However, it seems to follow from this case that the rational thought can go astray regardless of intelligence. Our results indicate that other aspects of rational thought, rather than intelligence, such as the ability or the motivation to notice incorrect intuitive inputs and overcome them, play a much more important role in the development and endorsement of foolish thoughts. Thus, it seems that Stanovich (2009) captured a real phenomenon when he coined the term “dysrationalia” - it indeed seems that people can hold irrational beliefs despite being reasonably intelligent.

However, before drawing stronger conclusions from these findings, few words of caution are needed. First, despite different aspects of rational thought being significant predictors of ESB, the complete model explained only about 20% of the variance in those beliefs. This means that some additional factors must play important role in explaining ESB. These factors can be related to rational thinking, but some other constructs could also be important. For example, in their Comprehensive Assessment of Rational Thinking (CART) battery, as components of rational thought, Stanovich et al. (2016) also measure constructs such as scientific reasoning, probabilistic numeracy or risk knowledge. They also suggest assessing additional cognitive styles such as Active Open-minded Thinking (e.g. Baron, Scott, Fincher, & Metz, 2015). Thus, these additional components of rational thought could perhaps explain some additional variance in ESB. In addition to these, there are several other factors that were shown to be important for these kinds of beliefs, such as locus of control (Groth-Marnat & Pegden, 1998; Tobacyk, Nagot, & Miller, 1988), and even socio-demographic, socio-economic and cultural background (Torgler, 2007). Therefore, although rational thinking is an important factor in explaining epistemically suspect beliefs, it might not be the only one, and possibly not even the most important one.

In addition to the variable choice, the important drawback of this study is the sample. Namely, only students of humanities and social sciences participated in this study (and mostly females). Narrow samples such as this one can be problematic for several reasons. The first is the range restriction. Although the width of the distribution of majority of the variables was satisfactory, the distribution of ESB was particularly narrow (see Figure A in Appendix) which could perhaps distort the effect sizes. Similarly, although the distribution of APM results is surprisingly wide given the student sample, it is nevertheless shifted towards higher values. It is thus possible that, in a more representative sample with a wider range of intelligence scores, intelligence would be significant predictor of ESB. Finally, using a student sample here may be an example of the conditioning on a collider. A collider is a variable that is related to both predictor and outcome. For example, being a student might be positively related with intelligence, and perhaps negatively with holding ESB. In cases like this, it is possible that controlling for a collider (whether statistically or by sampling) could distort and bias the relationship between the predictor and outcome (see Rohrer, 2018).

In conclusion, the current study fits well with Risen’s (2016) dual process model of superstitious beliefs which posits that System 1 provides intuitive, superstitious inputs, that, in order to dismiss superstitious thoughts, must be overridden by System 2 processing. However, we also show that not all aspects of System 2 processing are equally important. Namely, the ability and propensity to think rationally and analytically play an important role, whereas cognitive abilities do not. Therefore, our results suggest that “dysrationalia”, defined as holding irrational beliefs despite adequate intelligence, could be a real phenomenon. In other words, it is not about how intelligent someone is, but how willing and able one is to question the intuitive impulses, rather than uncritically embracing and accepting them. An intelligent person, but without a motivation or ability to reflect on his/her own intuitive ideas and beliefs, can nevertheless hold unsubstantiated beliefs.

These beliefs are sometimes just weird or funny, as in the case of a football player from the introduction, but other times can be quite serious and dangerous. Such is the case of the “Heaven’s gate” cult whose 39 members committed suicide in 1997 in order to reach the Hale-Bopp comet that was supposed to help them reach "the evolutionary level above human”. These individuals left behind a series of video interviews that make it clear that there was nothing wrong with their cognitive abilities. On the contrary, they were very thoughtful and reasoned, and seemed quite smart. Yet, they managed to hold such extreme irrational beliefs that ultimately led them to commit suicide. Thus, it is clear that people do not hold irrational beliefs primarily because they are not intelligent enough, but that the underpinnings of irrational thoughts are much more complex. Understanding more about motivational and cognitive underpinnings of irrational beliefs is important first step in reducing the prevalence of these beliefs or preventing people from embracing them in a first place. We believe that this study is a step in that direction.

## Figures and Tables

**Figure A:**
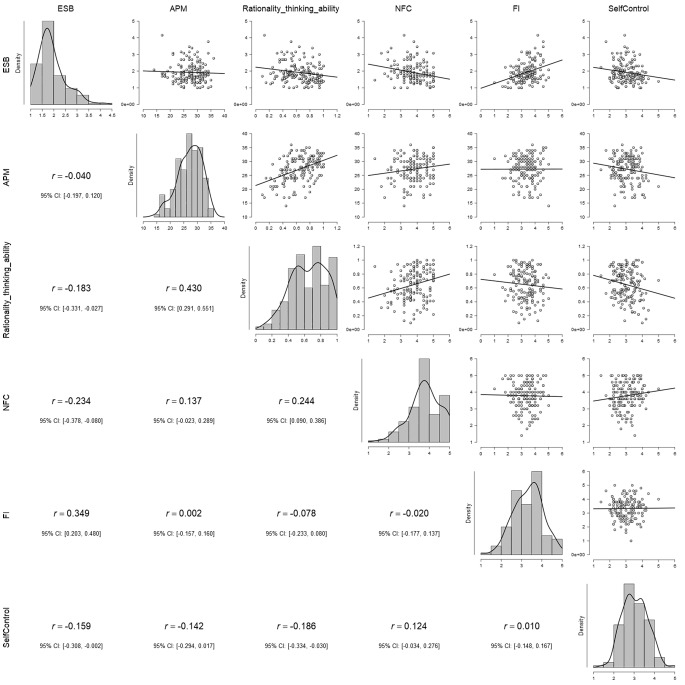
The distributions of the variables measured within the study, together with scatter plots and correlation coefficients representing the relationships between them. *Note*. ESB = Epistemically Suspect Beliefs; APM = Advanced Progressive Matrices; NFC = Need for Cognition; FI = Faith in Intuition.
